# Dentistry and the Dentists Bill

**Published:** 1921-06-25

**Authors:** 


### Dentistry and the Dentists Bill.

The new Dentists Bill, which has just been introduced into Parliament, has done at least one good thing—it has stimulated afresh a general discussion on the importance to the national health of the proper care of teeth. With regard to the Bill itself, it makes an important departure in the practice of dentistry, which, while it has aroused acute controversy in the profession, undoubtedly concerns very closely the public interest. The Bill, which is partly inspired by a desire to put dental practice more within the reach of the general public, will have the effect of admitting most of the unregistered dentists to the Dentists Register. The Dentists' Committee, of which the Chairman is Sir Frank Colyer, protests, in the name of 2,000 qualified men, that this proposal, if carried out, will in fact deprive the public of its principal safeguard against quackery—the power to distinguish between the qualified and the unqualified—and it has suggested as an amendment that a special list of unregistered men should be instituted, and that these should be given the distinctive title of "dental practitioner." Whether or not it is practicable or desirable to adopt this amendment, there is no doubt that a serious need exists for increasing the numerical strength of the dentists' profession, and for keeping its status high in order to attract men of the greatest ability.

The direct relationship between teeth and health is now more fully recognised than it has ever been before, but probably few people even now are aware that something like 80 per cent. of the adult population of this country have septic mouths. Many years ago Ruskin wrote (we believe to Mr. Edmund Gosse): "My £100 a year as Academician I spend in perfecting my collection of the palates of molluscs, who keep their inward economy as clean as a ship of the line. Few of your Academicians show an apparatus half so handsome when they open their mouths." If in those days the nation as a whole understood as clearly as Ruskin the importance of clean mouths, we should not now have to listen to the statements of insurance societies that neglect of teeth troubles is the cause of half the ill-health found among the industrial classes. Nor would the Chief Medical Officer of the Board of Education have to say that out of 6,000,000 children on the registers of elementary schools in England and Wales not less than half, or 3,000,000, needed dental treatment, and not less than 500,000 needed it urgently. According to some of the figures quoted by the medical correspondent of *The Times*, in some areas as many as 80 per cent. of the children aged from six to eight years require treatment, while in London 81 per cent. require treatment. The fact, however, that the Chief Medical Officer possesses the data which enable him to make these estimates and give these percentages is in itself a sign of progress. It is a sign of the extremely fine work done in the school clinics, under very difficult conditions, by qualified dentists, and it shows that at last this vital problem of health is being attacked in the right way and in the right place. We believe that the school clinic may prove to be the chief influence in giving the nation healthy teeth, not only by the practical preventive work which it may do, but by giving parents and the children themselves, and through them the next generation, a real sense of the importance of the care of the teeth from the earliest age. What is urgently required at the moment is an adequate supply of efficient dentists whose services may be at the command, because within the means, of every section of the public.

